# Chronic consumption of cassava juice induces cellular stress in rat substantia nigra

**DOI:** 10.22038/IJBMS.2019.38460.9131

**Published:** 2020-01

**Authors:** Christian de Jesús Rosas-Jarquín, Eduardo Rivadeneyra-Domínguez, Bertha Alicia León-Chávez, Rasajna Nadella, Aurora del Carmen Sánchez-García, Daniel Rembao-Bojórquez, Juan Francisco Rodríguez-Landa, Daniel Hernandez-Baltazar

**Affiliations:** 1 Posgrado en Neuroetología, Instituto de Neuroetología, Universidad Veracruzana. Xalapa, Veracruz. Mexico; 2 Facultad de Química Farmacéutica Biológica, Universidad Veracruzana. Xalapa, Veracruz. Mexico; 3 Facultad de Ciencias Químicas, Benemérita Universidad Autónoma de Puebla. Puebla, Puebla. Mexico; 4 IIIT Srikakulam, Rajiv Gandhi University of Knowledge Technologies (RGUKT); International collaboration ID: 1840; India; 5 Instituto Nacional de Neurología y Neurocirugía “Manuel Velasco Suárez”. Ciudad de México. Mexico; 6 Laboratorio de Neurofarmacología, Instituto de Neuroetología, Universidad Veracruzana, Xalapa, Veracruz. Mexico; 7 CONACyT-Instituto de Neuroetología, Universidad Veracruzana. Xalapa, Veracruz. Mexico

**Keywords:** Apoptosis, Inflammation, Hypertrophy, Manihot, Survival

## Abstract

**Objective(s):**

Cassava (*Manihot esculenta* Crantz) contains cyanogenic glycosides (linamarin and lotaustralin) that have been associated with neurological disorders in humans and rats. In basal ganglia, the dopaminergic neurons of substantia nigra* pars compacta* (SNpc) show high cytotoxic susceptibility; therefore, the chronic consumption of cassava (CCC) could induce neurodegeneration in SNpc. In this study we examine the impact of CCC on the integrity of the nigrostriatal system, including apoptosis and microgliosis.

**Materials and Methods:**

Male Wistar rats were administered cassava juice daily (3.57 g/kg and 28.56 g/kg, *per os*) or linamarin (0.15 mg/ml, IP), and its effects were evaluated in rota-rod and swim tests at days 7, 14, 21, 28, and 35 of administration. In SNpc, oxidative/nitrosative stress was determined by malondialdehyde/4-hydroxyalkenals (MDA-4-HAD) and nitrite contents. Tyrosine hydroxylase immunoreactivity (TH-IR) was evaluated in SNpc, neostriatum (NE), and nucleus accumbens (NA). Apoptosis and microgliosis were determined by active-caspase-3 (C3) and CD11b/c (OX42) expression in the medial region of SNpc.

**Results:**

Chronic administration of cassava juice, or linamarin, increased motor impairment. The rats that received 28.56 g/kg cassava showed increased MDA-4-HAD content in SNpc and nitrite levels in NE with respect to controls. Significant loss of TH-IR in SNpc, NE, and NA was not found. The 28.56 g/kg cassava administration produced dopaminergic atrophy and microgliosis, whereas linamarin induced hypertrophy and C3-related apoptosis in SNpc.

**Conclusion:**

CCC induces cellular stress on dopaminergic neurons, which could contribute to motor impairment in the rat.

## Introduction

Cassava (*Manihot esculenta* Crantz, belonging to Euphorbiaceae family) is cultivated in Latin America, Africa, and Asia (1). The plant is widely used in the food industry due to its high nutritional value (2-4); cassava intake in America is 105±330 g/day (5) and 940±777 g/day in Africa (6); however the cassava root has high levels of cyanogenic glycosides such as linamarin and lotaustralin, whose biotransformation can produce acetone cyanohydrin followed by cyanide (7, 8). Cassava leaves have a cyanide content ranging from 53 to 1,300 mg, while each kilogram of cassava root could contain 10 to 500 mg cyanide equivalents/Kg of dry matter (9). Increased concentrations of cyanide in blood could induce oxidative/nitrosative stress (ONS) due to mitochondrial dysfunction (10).

In humans, it has been described that chronic consumption of cassava (CCC) could cause neurological diseases such as Tropical Ataxic Neuropathy (TAN) (11, 12) and Konzo (13-15), which are characterized by motor impairment and cognitive dysfunction (16). For TAN, a peripheral polyneuropathy, spinal cord demyelination has been suggested; in Konzo disorder the upper motor neuron lesions are related (17, 18). To date, there exists only little evidence of neurobiological basis of these disorders. 

In this context, murine models have been used to elucidate the cellular mechanisms that underlie neurological damage caused by cassava. The motor impairment due to cassava juice consumption or intraperitoneal administration of cassava toxic compounds (linamarin/acetone cyanohydrin) has been widely reported (1, 19-21). Interestingly, the chronic consumption of cassava induces multiple cellular damages. The oral consumption of cassava for 30 days produces spongiosis increase in the lateral geniculate body and hypertrophy of superior colliculus nucleus (22). The administration of toxic compounds also gives relevant findings; the oral administration of 20 mM acetone cyanohydrin during 42 days caused neurodegeneration in the thalamus, paraventricular nucleus, dorsal endopiriform, and lateral entorhinal regions in the cerebral cortex (23). Likewise, the intrahippocampal administration of acetone cyanohydrin induced loss of CA1 hippocampal neurons at day 7 post-lesion (20). Recently, a study reported that cassava produces significant increase in the levels of serum glucose, alanine aminotransaminase, aspartate aminotransaminase, and alkaline phosphatase (24). In addition, the oral administration of thiocyanate for a period of 5 days produced carbamoylation of basic myelin protein (BMP), filamentous actin protein (FAP), and glial fibrillary acidic protein (GFAP), as well as differential expression of proteins that are involved in the oxidative mechanisms (peroxiredoxin 6), endocytic vesicular trafficking (dynamin), protein folding process (disulfide isomerase), and cytoskeleton maintenance (α-spectrin) (25). Additionally, evidence of cell death (necrosis) in an *in vitro *study has been identified (26). On the whole, the evidence shows that the toxic compounds of cassava induce cellular stress and neurodegeneration.

Actually no reports exist about the evaluation of the neurodegeneration in specific brain circuits of motor control such as the nigrostriatal pathway. The nigrostriatal pathway, integrated by the substantia nigra* pars compacta* (SNpc), medial forebrain bundle, and neostriatum (NE), modulate the motor activity through the dopamine released from dopaminergic neurons (DN) that send afferents mainly to NE, ventral tegmental area (VTA), and the cerebral cortex (27). The DN are highly susceptible to ONS due to low levels of glutathione peroxidase and high concentrations of Fe^+^ and free radicals, which favor neuromelanin deposits; the cellular stress produced finally blocks the natural detoxification properties of DN (28, 29). As a consequence of the DN degeneration, the involvement of microgliosis and cell death occurs, which could explain the progressive motor impairment related to cassava injection.

In this study, the cytotoxic effect of cassava on nigral DN was determined. The knowledge of histopathological features such as degeneration, microgliosis, and apoptosis, induced due to CCC, is the key to understanding the neurobiological effects of cassava-related human diseases.

## Materials and Methods

***Ethics***

Entire experimental procedures were performed according to the International Guide for the Care and Use of Laboratory Animals (National Institute of Health, 1999), and ethics statements from Universidad Veracruzana (NOM-062-ZOO-1999 and NOM-087-ECOL-SSA1-2002). Considering the ethical recommendations for preclinical research, the relatively low number of animals that were included in the present study is consistent with the 3Rs (30) and meet the minimum required to perform statistical analysis. All efforts were made to minimize animal discomfort during the study. 

***Animals***

Thirty adult male Wistar rats (250–300 g) were included in the study. The rats were housed in Plexiglas cages (n = 3 to 4 per cage) under a 12/12 hr light/dark cycle (lights on at 7:00 AM) at room temperature (RT) with water and food *ad libitum.*

***Experimental groups***

The rats were distributed into four groups, Vehicle (0.2 ml of water, *per os (PO)*; n=8, LOW Dose-cassava juice (LD-cassava, 3.57 g/kg), High Dose-cassava juice (HD-cassava, 28.56 g/kg), *PO,* n=8, and Linamarin (0.15 mg/ml, IP, n=6) as pharmacological controls. The dosages were calculated considering that a person with a weight of 70 Kg consumed approximately 500 g of cassava per day (5), which would correspond to 7.14 g of cassava per Kg of bodyweight. According to this rationale and previous reports of behavioral effect of cassava in rats (1), LD and HD-cassava dosages correspond to 0.5X and 4X of the reference dose (7.14 g/kg for 2 ml water), respectively. In order to ensure efficient fluid intake, reduced stress and less esophageal damage in all groups, the administration of water, cassava juice, or linamarin was done every 24 hr for 35 days through a displaceable sterilized intraesophageal polyethylene cannula (4 cm length, 1.0 mm diameter; S-54-HLCole-Parmer, Vernon Hills, IL, USA) coupled to a disposable syringe (Roger, Chalco, Estado de México, México) according to previous reports (21, 31). The weight of all rats was recorded every 48 hr. The animal survival percentage was determined at the end of the study.

***Cassava juice extraction***

Cassava tubers were harvested at the fall season in the Municipality of Tierra Blanca, Veracruz, Mexico (18.4458° N, 96.3598° W). The authentication of the Cassava plant and its tubers was performed in the Instituto de Ecologia A.C. of Xalapa, Veracruz, Mexico (reference # 006186). The method for extraction of cassava juice has been previously described (21). Briefly, fresh juice was prepared daily by homogenizing the pieces and filtration. 

***Behavioral tests***

All behavioral paradigms were performed every 7^th^ day for a period of 35 days in total (n = 6-8 *per* group).

*Rotarod*

In this test, motor coordination has been assessed based on the variable latency to fall at 18 RPM (1, 21). The rats received previous training (1–5 min) at increasing speed (5 to 18 RPM) for a period of 5 days before the experiment day (32). The training was given solely on the previous day, mentioned as day 0.

*Swimming test*

This test was used to evaluate the number of spins, referred to as the periods during which the rat was spinning rather than moving forward. All experimental sessions were video-recorded by a Nikon Coolpix s5200 camera. Two independent blind watchers analyzed each video, and the records were performed using *ex professo* software.

***Oxidative/nitrosative stress (ONS)***

Lipoperoxidation assay and nitrites content determination were performed, as mentioned elsewhere (33). Briefly, the animals (n = 3–4 *per* group) were euthanized with overdose of sodium pentobarbital (100 mg/kg). The whole nucleus of interest (SNpc and neostriatum) was collected by microdissection from fresh brain tissue. Twenty milligrams of SNpc and 60 mg of the neostriatum were homogenized with 300 μl and 500 μl of 0.1 M phosphate buffer solution (0.1 M PBS) at pH 7.4, respectively, followed by centrifugation at 12,500 RPM during 30 min at 4 °C. Malondialdehyde/4-hydroxyalkenals (MDA-4-HAD) was quantified by Gerard-Monnier assay at 586 nm; the nitrites content was measured through the Griess colorimetric reaction at 540 nm using a spectrophotometer (SmartSpec 3000; BioRad, USA). The values obtained were normalized in terms of protein concentrations determined by Biuret´s method. 

***Collection of brain tissue***

A total of four rats per group were euthanized and perfused transcardially with 100 ml of 0.1 M PBS, followed by 50 ml of 4% paraformaldehyde (PFA) prepared in 0.1 M PBS, using a peristaltic pump. The brain was extracted and kept in 4% PFA for fixation for 24 hr, and later transferred to 30% sucrose solution in 0.1M PBS. The brain was sectioned in a coronal plane at 35 μm using a freezing sliding microtome (Leica CM1520, Germany). According to Atlas of Paxinos and Watson (2007), the interaural coordinates for each brain nucleus include: Whole SNpc (from 4.44 mm to 2.40 mm), medial SNpc /VTA (4.20 mm to 3.72 mm), whole neostriatum (12.24 mm to 7.56 mm) and neostriatum/accumbens nucleus (12.24 mm to 9.60 mm). 

The separation of SNpc and VTA by the medial terminal nucleus of the accessory optic tract (interaural 3.80 mm) helped us in the identification and extraction of medial SNpc. The evaluation of TH-IR was focused mainly in the region of ventrolateral neostriatum because this region receives afferents mainly from SNpc. 

A total of 50–60 slices of the whole SNpc and 75–80 slices of neostriatum/accumbens nucleus were collected, distributed in 6 batches, each one containing 9 to 10 slices (SNpc) and 11 to 12 slices for neostriatum/accumbens nucleus, which constituted a representative sample of all levels, i.e., around 20% tissue each from rostral, medial, and caudal regions of bilateral SNpc or neostriatum/accumbens nucleus (34). The slices were stored in a cryoprotective solution (glycerol 30% and ethylene glycol 30% in 0.5 M PBS pH 7.4). 

***Immunostaining***

*Immunohistochemistry revealed with diaminobenzidine*

After washing the tissues with 0.1M PBS, they were subjected to the inactivation of endogenous peroxidase with 3% hydrogen peroxide and 10% absolute methanol in 0.1 M PBS. Non-specific sites were blocked for one hour with 10% normal horse serum (Vector Laboratories, USA) prepared in 0.3% PBS-Triton. Incubation was performed with monoclonal anti-tyrosine hydroxylase (TH) generated in mouse (1:1000; Cat. T1299, Sigma-Aldrich, USA) for 24 hours at 4°C. Then the sections were incubated for 2 hr at RT with biotinylated anti-mouse IgG produced in horse serum (1:200; Cat. BA-2000 Vector Laboratories, USA). Subsequently, it was incubated with an avidin-biotin-peroxidase complex kit (ABC; Vector Laboratories, USA) at RT for 2 hr, and then washed with 0.1 M PBS and incubated with 300 μl of 3, 3´-diaminobenzidine solution (DAB kit, Vector Laboratories, USA). The tissues were then mounted on poly-L-lysine-treated slides (Sigma Aldrich, USA) and were finally covered with Entellan (Merck, San Francisco, CA, USA) and a coverslip. 

In this study a representative sample of TH-immunoreactive (TH-IR) cells that were distributed only in the medial region of left SNpc. The number of cells was quantified and is expressed as an average in each group. The size of the soma of nigral dopaminergic neurons and TH-IR arborizations in neostriatum and nucleus accumbens were studied with optical density from ImageJ 1.50i software. 

*Immunofluorescence for TH, cleaved-caspase-3, and microglia*

The single or double immunostaining was performed according to the previously described protocol (34, 35). Briefly, 35 μm coronal sections of the SNpc were used. The slices were permeabilized with 0.1 M PBS containing 0.1% Triton, and non-specific binding sites were blocked with 10% horse serum (Invitrogen, USA) in 0.1% PBS-Triton for 1 hr at RT. 

For incubation, the following primary antibodies were used, monoclonal anti-TH made in mouse (1:1000; Cat.T1299, Sigma-Aldrich, USA), polyclonal anti-TH produced in rabbit (1:1000; Cat. ab6211, Abcam, USA), polyclonal anti-cleavage-caspase-3 made in rabbit (1:600; Cat. C8487, Sigma-Aldrich, USA) and monoclonal anti-CD11b/c (OX42) produced in mouse (1:500; Cat. ab1211, Abcam, USA). Secondary antibodies used in this study include Alexa 488 goat anti-rabbit IgG (1:200; Cat. ab150077, Abcam, USA), Alexa 488 donkey anti-mouse IgG (1:200; Cat. ab150105, Abcam, USA), Texas Red anti-IgG rabbit made in goat (1:300; Cat. TI1000, Vector Laboratories USA) and Texas Red anti-mouse IgG made in horse (1:300; Cat. TI2000, Vector Laboratories, USA). The nuclei were counterstained with 1 μM Hoechst 33342 (Sigma Aldrich, USA). Negative controls of the technique did not receive any primary antibody. 

As a positive control, 2 μl of 50 nM staurosporine (stau; Sigma Aldrich, USA), an apoptosis inducer, was used. Staurosporine was administered in the SNpc by stereotaxic procedures under anesthesia (Ketamine/Xylazine) contemplating the coordinates, anterior-posterior +2.4 mm from the interaural midpoint; medial-lateral +1.8 mm of the intraparietal suture; dorsal-ventral -6.9 mm of the dura mater. The toxin was injected with a flow of 0.2 μl/min with micropump (Stoelting; USA) (34). 

Image acquisition was performed with a Leica DM1000LED/DFC450C epifluorescence microscope (Nussloch, Germany) at emission/excitation wavelengths of 405/465 nm for the blue channel, 488/522 nm for the green channel, and 568/635 nm for the red channel. 

For this study, a representative sample (~9%) of TH-IR cells present in the medial region of left SNpc was analyzed by densitometry (ImageJ software), and the results were graphed using GraphPad Prism 5.0 (GraphPad Software Inc., La Jolla, CA, USA).

***Statistical analysis***

The data are presented as the mean±standard error. The values obtained in the behavioral tests and bodyweight measurements were analyzed by two-way ANOVA considering two factors (days, treatments) and their interactions. For values of ONS levels, cell counting, and optical density one way ANOVA followed by Newman-Keuls *post hoc* test (*P*≤ 0.05) was used.

## Results

***Survival and bodyweight***

The cassava administration induced survival of 97%. All rats showed a gradual increase in their bodyweight from day 0 to day 35 in each group (Table 1). When compared to day 7, the weight gain of the rats at day 35 was almost the same (~58 g) in both vehicle (Veh) and linamarin (Lin) groups. Similarly, the weight variation between day 35 and day 7 in HD-cassava is higher (~63 g), and LD-cassava is lower (~53 g). Overall, the weight gain difference between day 7 and day 35 of HD-cassava is highest among all experimental groups (Table 1), indicating a significant gain in bodyweight [F_(6,173)_=59.11; *P*<0.05]. However, there are no differences between treatments [F_(4,173)_=0.67; *P*=0.571] and interactions [F_(18,173)_=0.55; *P*=0.930].

***Motor impairment***

**Figure 1:**
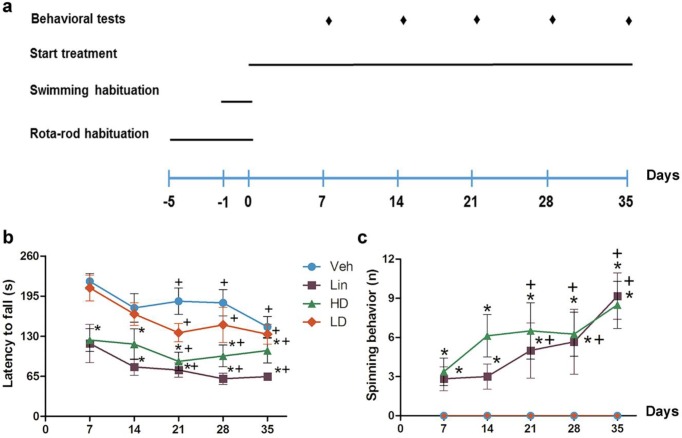
Motor impairment induced by chronic consumption of cassava juice. a) Experimental design. b) Rota-rod test. c) Swim test. * *P*<0.05 vs vehicle, + between days of treatment. Veh: vehicle; Lin: linamarin; HD: high dose; LD: low dose; s: seconds; n: number

Motor activity was assessed by Rota-rod and swim tests every 7^th^ day for 35 days (Figure 1 a). Regarding the variable latency to fall (Figure 1 b), LD and HD-cassava juice significantly decreased motor coordination *vs* vehicle. The linamarin group showed decreased motor coordination when compared to HD-cassava [F_(3,144)_= 27.14; *P*<0.001]. In all experimental groups, motor impairment was present from day 21 post-treatment. The vehicle group maintained the tendency to avoid falls [F_(4,144)_= 4.771; *P*<0.001]. However, no interaction was found between the variables, treatment, and days of treatment [F_(12,144)_= 0.488; *P*=0.918]. In the swim test (Figure 1 c), the HD-cassava and linamarin groups showed significant increase in the variable spinning behavior along the evaluation time course. Linamarin treatment produced greater motor impairment than the HD-cassava.

***Cytotoxic role of Cassava in SNpc, neostriatum, and accumbens***

**Figure 2:**
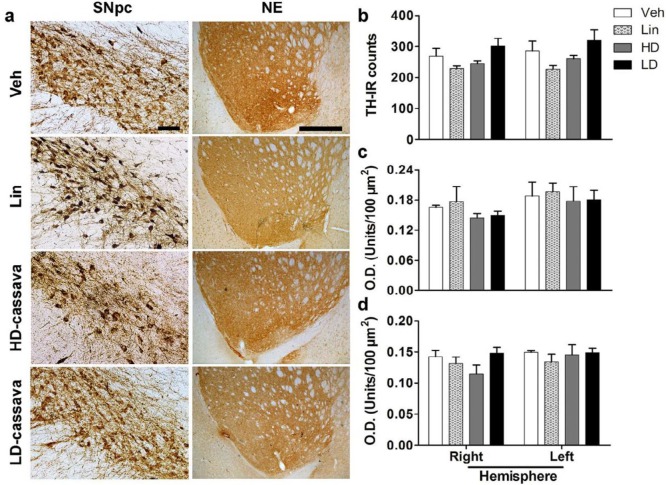
Evaluation of TH content in the left hemisphere of medial SNpc, ventrolateral neostriatum (NE), and nucleus accumbens. a) Immunodetection of TH in SNpc and NE. Scale bar= 100 μm (SNpc) and 200 μm for NE images. b) Cell counting of TH-IR cells in SNpc. c) Levels of TH-IR in NE. d) Levels of TH-IR in nucleus accumbens. TH: Tyrosine hydroxylase; NE: Neostriatum; O.D.: Optical density

According to TH-IR counting from both sides of SNpc, any experimental condition showed significant decrease in the total number of DN (Figures 2 a and b); however the tendency is low in HD-cassava and linamarin groups in HD-cassava and linamarin groups. In the neostriatum, the optical density did not reveal any significant differences between treatments vs vehicle (Figure 2 c). At the same time, no significant variation was found in TH levels in nucleus accumbens (Figure 2d). Regarding the DN population, the histological evidence and TH quantifications indicate that cassava juice or linamarin did neither decrease nigral dopaminergic population nor nuclei projection in neostriatum and accumbens nucleus.

In order to determine the cytotoxicity of cassava juice or its toxic compound (linamarin), neostriatum and SNpc were evaluated by ONS assays (lipoperoxidation and nitrite contents) and immunotechniques to identify the neurodegeneration features, microgliosis, and C3-related apoptosis. 

**Figure 3:**
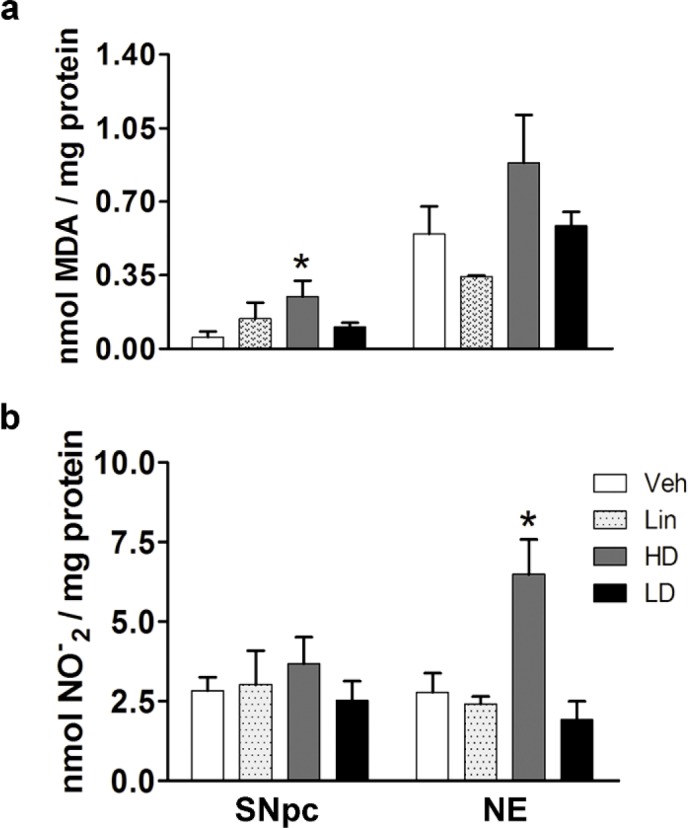
Determination of oxidative/nitrosative stress in unilateral SNpc and neostriatum (NE). a) MDA-4-HAD levels. b) Nitrite content. * P<0.05 vs vehicle. Veh: vehicle; Lin: linamarin; HD: high dose; LD: low dose; SNpc: Substantia nigra pars compacta; NE: Neostriatum; MDA-4-HAD: Malondialdehyde/4-hydroxyalkenals

The determination of ONS was performed in both sides of SNpc and NE; however, no significant difference was found. For this reason, we only showed one side of each nucleus (left side, Figure 3). In SNpc, HD-cassava increased MDA-4-HAD levels 6.45X with respect to the vehicle group (Figure 3 a). In neostriatum, HD-cassava showed an increase of 2.03X in nitrites content when compared with vehicle (Figure 3 b). The increase of MDA levels in SNpc is related to nitrites content in neostriatum from same cerebral hemisphere.

**Figure 4:**
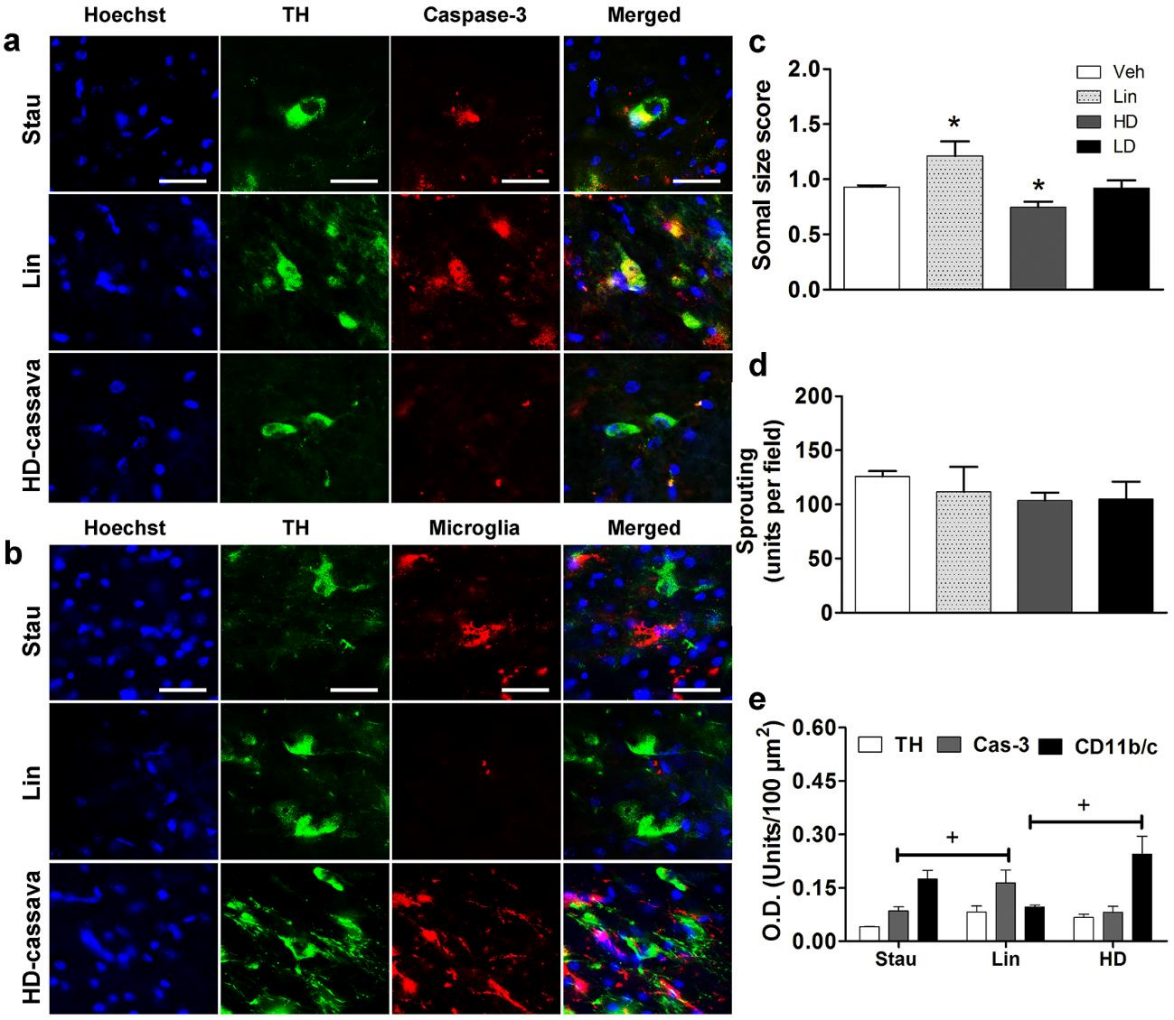
Apoptosis and microgliosis in medial SNpc (unilateral). a) Immunolabeling of caspase-3. b) Microglia around TH-IR neurons (green channel). Caspase-3 and CD11b/c (microglia marker) are shown in the red channel. Scale bar = 20 μm. Graphs showing the soma size (c), neuronal sprouting (d), and comparative levels of TH, caspase-3 (Cas-3), and CD11b/c-positive cells in all treatments (e). O.D.: Optical density. * *P*<0.05 vs vehicle, + treatment vs treatment

Immunostaining with TH / OX-42 and TH / Caspase-3 antibodies was performed to know if the cellular stress ends with microgliosis and apoptosis in SNpc. The results show that Stau induced caspase-3 expression in DN and microgliosis as expected (Figures 4 a and b). The administration of linamarin induced high expression of caspase-3 in TH-IR neurons (0.16 units per 100 μm^2^) and low reactive microgliosis (0.09 units per 100 μm^2^). In contrast, the HD-cassava showed low expression of caspase-3 in TH-IR cells (0.06 units *per* 100 μm^2^), but high reactive microgliosis (0.28 units *per* 100 μm^2^) (Figure 4 e). Interestingly, the treatment with linamarin increased cell size (1.211±0.065) reflected hypertrophy, the HD-cassava decreased in size (0.721±0.028) and showed atrophy when compared with the vehicle condition (0.931±0.014) (Figure 4 c). No significant differences were found in TH content, sprouting neurons in all groups *vs* vehicle (Figures 4 d and e).

## Discussion

In this study we found that the chronic consumption of cassava did not affect the survival and bodyweight of the rats over 35 days, which is related to the nutrimental contribution of cassava (3). However, at the cellular level, our results together give evidence that chronic consumption of HD-cassava juice or a toxic compound (linamarin), for 35 days induces changes in cell size (hypertrophy or atrophy), C3-related apoptosis, and microgliosis in SNpc, without significant damage in dopaminergic population or dopamine innervation in neostriatum. These cellular events could contribute to motor impairment in Wistar rats associated with cassava juice consumption. 

Analyzing the motor test, the fact that LD-cassava, HD-cassava, and linamarin groups showed decrease in their latency in the roller when compared with the vehicle group is highly expected (1, 21); the participation of cerebellar and spinal cord associated with this behavior was discarded because the rat can execute its movements correctly and does stay on the roller (36, 37). In other fields, the swim test gave evidence that the HD-cassava and linamarin treatments induce spinning behavior as a result of motor dysfunction; this finding is in accordance with previous reports of chronic consumption of cycad seeds or cassava juice in a rat model (1). The behavioral features displayed with cassava consumption suggest the involvement of nigrostriatal pathway. 

In this study, the number of dopaminergic neurons in SNpc was not decreased, but cellular adaptation in SNpc after chronic cassava consumption was evidenced. Through the use of neurotoxins such as 6-hydroxydopamine and paraquat, it is possible to identify that dopaminergic neurons of the SNpc are highly susceptible to exogenous toxins (38). Therefore, when adding potentially toxic compounds, this susceptibility could be increased until they cause degeneration, cellular adaptation, or death of dopaminergic neurons in SNpc. In this study, two cytotoxic effects of cassava were determined. 

First, the HD-cassava juice induced high levels of nitrites in NE accompanied by an increase in lipoperoxidation levels, cellular atrophy, microgliosis and absence of C3-related apoptosis in SNpc. Atrophy is a cellular adaptation characterized for significant decrease of cellular size (39); this phenomenon is associated with ONS. The presence of high levels of ONS is not always an indicative of cell degeneration, because several cellular processes require the oxidation-reduction balance generated by free radicals (40); however, the determination of ONS may give an overview of the biochemical state in early stages of chronic pathologies (41, 42). In this context, our results show that HD-cassava consumption *vs* control induced unilateral high levels of MDA-4-HAD (in SNpc) and nitrites (in NE), which is an evidence of the cytotoxic effect of CCC. After cassava consumption, increase of cyanide levels, a toxic compound of cassava juice, in blood could increase free radicals and exacerbate the use of exogenous NO^-2^ into mitochondria promoting increased lipoperoxidation products. The accumulation of high levels of MDA-4-HAD in DN can alter the respiratory mitochondrial chain by binding of cyanide to the binuclear hematic center of cytochrome C oxidase and produce cell damage (43). 

On the other side, the presence of high levels of nitrite contents in NE could be a consequence of damage in the nigrostriatal circuit derived from DN atrophy. This adaptive process can lead to nitrite accumulation in NE by nitric oxide synthase (NOS)/nitric oxide (NO) deregulation. Other sources of nitrite ions are the capillary vasodilation and microglia recruitment as a result of DN degeneration (44), which is not observed in our experimental conditions; interestingly high levels of microglia were determined in HD-cassava treatment. If microgliosis does not always relate to cytotoxicity, in our study, similar levels of caspase-3 in HD-cassava and Stau groups reflect the presence of mild cytotoxic effect of HD-cassava treatment. However, this condition did neither favor significant damage in the number of dopaminergic neurons nor loss of dopamine innervation in neostriatum. For this, we suppose that the high presence of microgliosis is related to the neuroprotective role of microglia. 

In the linamarin group, several histopathological features were found. In SNpc and NE, no significant changes of MDA-4-HAD, nitrite contents, necrotic bodies, or microgliosis were observed. In this study, the linamarin dose used causes neuronal hypertrophy and high caspase-3 expression levels in TH-IR neurons of SNpc with respect to stau or vehicle groups. In our experimental conditions, neuronal hypertrophy could be the result of transient changes of free radical concentration in the microenvironment of the dopaminergic neurons. The free radicals can act on the membrane lipids by decreasing their fluidity, the reduction of electrochemical potential and increase in membrane permeability leading to neuronal hypertrophy (45, 46). This type of cellular adaptation is characterized by the cytoplasmic accumulation of ions, free radicals, proteins, and carbohydrates into cell; when the cell osmolarity is disrupted, neuronal death occurs by an increase in the activity of calcium channels, dysfunctional proteolysis, alpha-synuclein aggregation, and the formation of mutant proteins can occur (47). The cyanogenic nature of linamarin could favor glutamate-related excitotoxicity and consequently caspase-3-related apoptosis, as was identified in this work (48). Interestingly, our study showed expression of caspase-3 in dopaminergic neurons of SNpc sampled (9%) without significant microgliosis or significant loss of dopaminergic population in SNpc, which is supposed to cause a discrete neuronal damage due to HD-cassava treatment. 

**Table 1:** Bodyweight variation among the experimental groups

**Days**	**Vehicle**	**Lin**	**Cassava ** **juice**	
		**0.15 mg/ml**	**HD (28.56 g/Kg)**	**LD (3.57 g/Kg)**
0	291.14±4.86	293.00±7.67	279.75±383	283.00±3.91
7	306.57±6.07*	307.33±8.04*	295.25±4.58*	299.75±8.61*
14	323.71±7.24 ^&^	321.67±7.46 ^&^	313.00±4.57 ^&^	310.00±7.12 ^&^
21	339.14±5.89 ^&^	335.33±7.94 ^&^	329.25±4.91 ^&^	324.25±7.85 ^&^
28	351.14±6.46 ^&^^+^	348.67±7.69 ^&^^+^	342.50±4.77 ^& ^^+^	338.25±7.02 ^& ^^+^
35	364.29±6.99 ^&^^+^	365.33±8.46 ^&^^+^	358.25±4.89 ^& ^^+^	352.50±7.43 ^& ^^+^

* *P*<0.05 vs day 0 of treatment; & *P*<0.05 vs days 0 and 7 of treatment; +*P*<0.05 vs day 14 of treatment

Lin: linamarin; HD: high dose; LD: low dose

## Conclusion

The chronic consumption of cassava does neither favor loss of bodyweight nor negative survival in Wistar rats. The consumption of a daily dose of 28.56 g/kg of cassava juice for 35 days increased nitrite content and lipoperoxidation levels, dopaminergic atrophy, and microgliosis in SNpc. The administration of linamarin induced hypertrophy and caspase-3 mediated apoptosis. Cellular damage and microgliosis can contribute partially to motor impairment in male Wistar rats associated with cassava consumption. 
